# Perforated Ileal Diverticulitis Presenting With Acute Peritonitis in an Elderly Patient: A Case Report

**DOI:** 10.7759/cureus.108983

**Published:** 2026-05-16

**Authors:** Hugo E Mora Moreno, Melba Rivera Felix, Alondra Y Ramirez Gomez, Javier Querea Vázquez, Grecia Z Ponce Otero

**Affiliations:** 1 General Surgery, Hospital General Dr. Miguel Silva, Morelia, MEX

**Keywords:** acute peritonitis, ileal diverticulitis, perforated ileal diverticulum, segmental ileal resection, small-bowel diverticulosis

## Abstract

Ileal diverticulitis is an uncommon cause of acute abdomen, and perforation is a rare but serious complication that may lead to secondary peritonitis. Its clinical presentation is often nonspecific and may mimic more common intra-abdominal emergencies, making computed tomography (CT) essential for diagnosis. We report the case of a 79-year-old man who presented with a three-day history of diffuse abdominal pain, nausea, vomiting, inability to tolerate oral intake, and absence of bowel movements and passage of flatus. Physical examination revealed abdominal distension, diffuse tenderness, guarding, and signs of peritoneal irritation. Laboratory studies showed mild leukocytosis and markedly elevated C-reactive protein. Contrast-enhanced CT demonstrated multiple ileal diverticula, segmental bowel wall thickening, inflammatory changes, pneumoperitoneum, and an adjacent fluid collection, consistent with perforated ileal diverticulitis and secondary peritonitis. Exploratory laparotomy revealed multiple ileal diverticula involving approximately 50 cm of the distal ileum, purulent and enteric contamination, and a perforated ileal diverticulum measuring approximately 5 mm. Segmental ileal resection with creation of an end ileostomy was performed. Histopathological examination confirmed acute diverticulitis with diverticular perforation and no evidence of malignancy. The patient had a favorable postoperative course and was discharged on postoperative day 7. This case highlights the importance of considering ileal diverticulitis in elderly patients with acute peritonitis and emphasizes the role of CT and timely surgical management in complicated cases.

## Introduction

Small-bowel diverticula are uncommon gastrointestinal lesions that are most frequently reported in older adults. Most are acquired pseudodiverticula that develop at points of relative weakness in the bowel wall, particularly along the mesenteric border where blood vessels penetrate the muscular layer. Although many cases remain asymptomatic and are discovered incidentally during imaging, surgery, or autopsy, symptomatic disease may present with vague abdominal symptoms and can be associated with clinically significant complications, including diverticulitis, hemorrhage, obstruction, abscess formation, and perforation [1].

The diagnosis of small-bowel diverticulitis is challenging because its clinical manifestations often overlap with more common causes of acute abdomen, such as appendicitis, colonic diverticulitis, Crohn's disease, infectious ileitis, bowel obstruction, and perforated viscus from other etiologies. Cross-sectional imaging may demonstrate small-bowel diverticula, focal or segmental bowel wall thickening, surrounding inflammatory changes, localized fluid collection, abscess formation, extraluminal gas, or pneumoperitoneum when perforation has occurred [2].

Ileal diverticulitis is particularly rare and may be difficult to distinguish from other inflammatory conditions affecting the distal small bowel and presenting with right lower quadrant symptoms. Because of its nonspecific presentation, it may be misdiagnosed as acute appendicitis, terminal ileitis, Crohn's disease, or infectious enteritis, especially when abdominal pain and inflammatory markers are the predominant findings [3,4]. Perforation of an ileal diverticulum is an uncommon but serious complication that may result in enteric contamination, secondary peritonitis, and the need for urgent surgical management [5].

We report the case of a 79-year-old man with perforated ileal diverticulitis presenting with acute peritonitis. The diagnosis was supported by contrast-enhanced computed tomography (CT) findings and confirmed intraoperatively and histopathologically. The patient was managed with segmental ileal resection and the creation of an end ileostomy. This report illustrates an uncommon cause of secondary peritonitis and underscores the need to include complicated ileal diverticular disease in the differential diagnosis of acute abdomen in older adults.

## Case presentation

A 79-year-old man presented to the emergency department with a three-day history of abdominal pain. The pain had a gradual onset, was continuous and diffuse, and progressively increased in intensity. It was associated with nausea, vomiting, inability to tolerate oral intake, and absence of bowel movements and passage of flatus. The patient denied fever, weight loss, night sweats, chest pain, cough, dyspnea, or other symptoms. His medical history was significant for hypertension diagnosed six years earlier, treated with losartan 50 mg once daily. He had no prior surgical history. He denied tobacco use, alcohol consumption, and illicit drug use. His family history was negative for diverticular disease, inflammatory bowel disease, colorectal cancer, or other known gastrointestinal disorders.

On physical examination, his vital signs were as follows: blood pressure, 129/72 mmHg; heart rate, 78 beats per minute; respiratory rate, 20 breaths per minute; temperature, 37°C; and oxygen saturation, 95% on room air. The patient was alert and oriented. Abdominal examination revealed distension, diffuse tenderness, guarding, and signs of peritoneal irritation. Given these findings, the general surgery team was consulted on the same day of presentation, and the patient was admitted for further evaluation and management.

Laboratory studies showed mild leukocytosis, markedly elevated C-reactive protein, mild prolongation of prothrombin time, elevated international normalized ratio, and mild aspartate aminotransferase elevation (Table 1).

**Table 1:** Laboratory findings on admission. Initial laboratory evaluation demonstrating mild leukocytosis, markedly elevated C-reactive protein, mild coagulation abnormalities, and mild aspartate aminotransferase elevation.

Test	Result	Reference range
White blood cells	10.8 × 10³/µL	4.5-10 × 10³/µL
Hemoglobin	15.3 g/dL	13-17 g/dL
Platelets	289 × 10³/µL	150-400 × 10³/µL
Prothrombin time	18.8 sec	12.5-16.5 sec
Partial thromboplastin time	30.5 sec	24.8-34.4 sec
International normalized ratio	1.44	0.8-1.3
Glucose	99 mg/dL	74-109 mg/dL
C-reactive protein	417 mg/L	0-6 mg/L
Aspartate aminotransferase	45 U/L	0-40 U/L
Alanine aminotransferase	15 U/L	0-41 U/L
Lactate dehydrogenase	264 U/L	120-300 U/L

**Figure 1:**
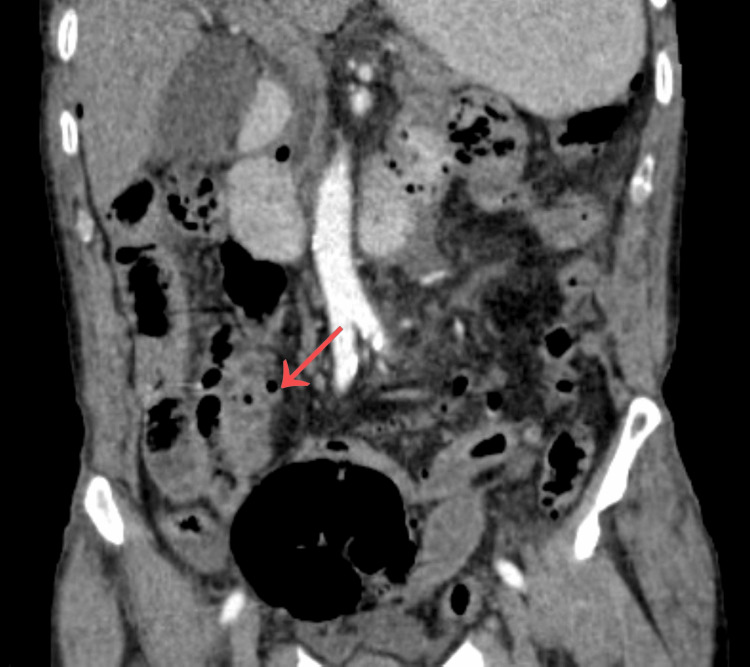
Ileal diverticula with inflammatory changes on computed tomography. Coronal contrast-enhanced computed tomography image demonstrating ileal diverticular disease. The ileal diverticular outpouching is indicated by the arrow, with adjacent segmental bowel wall thickening and surrounding inflammatory fat stranding corresponding to the inflammatory changes described in the text.

**Figure 2:**
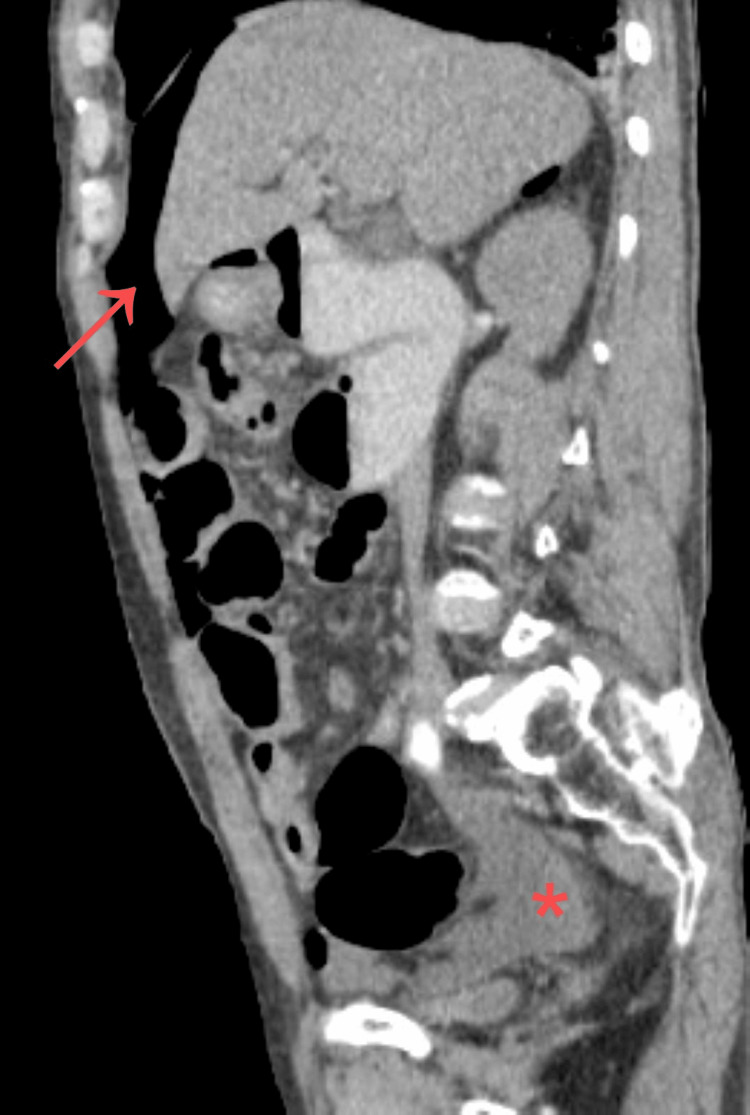
Pneumoperitoneum and pelvic fluid collection. Sagittal contrast-enhanced computed tomography image showing anterior pneumoperitoneum, indicated by the arrow, and an associated pelvic fluid collection, indicated by the asterisk. These findings support the diagnosis of perforated ileal diverticulitis with secondary peritonitis.

Contrast-enhanced CT of the abdomen demonstrated multiple ileal diverticula, most evident in the distal ileal loops, with adjacent segmental bowel wall thickening and surrounding inflammatory fat stranding (Figure 1). Pneumoperitoneum and an adjacent intra-abdominal fluid collection were also identified (Figure 2), supporting the diagnosis of perforated ileal diverticulitis with secondary peritonitis. No evidence of bowel obstruction or ischemia was observed elsewhere in the small or large bowel.

**Figure 3:**
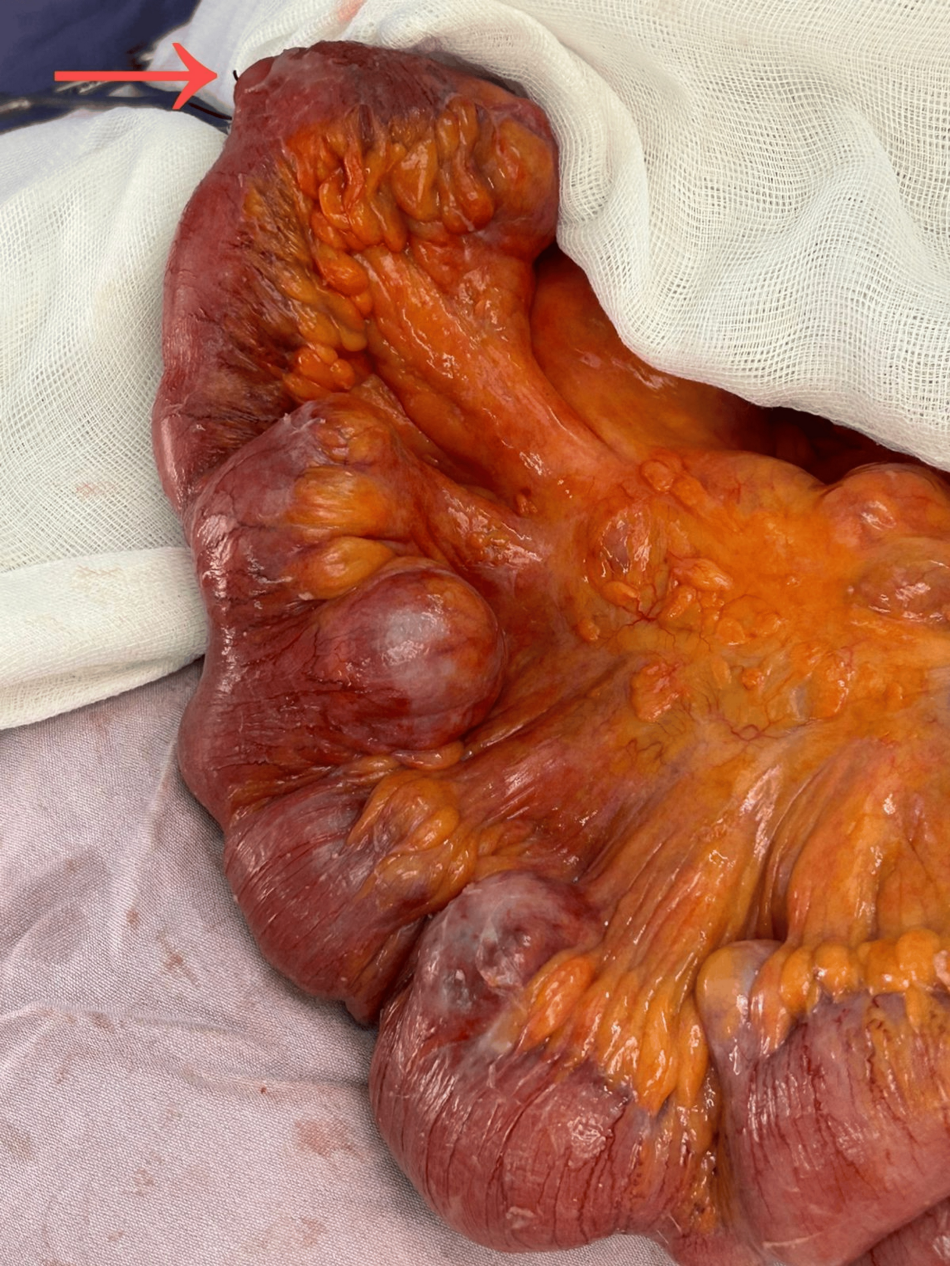
Intraoperative view of a perforated ileal diverticulum. Intraoperative photograph showing multiple diverticula along the affected ileal segment. The arrow indicates the diverticular perforation site, which corresponded to the source of enteric contamination and secondary peritonitis.

Given the clinical and imaging findings, an exploratory laparotomy was performed. Through a supra- and infraumbilical midline incision, multiple ileal diverticula involving approximately 50 cm of bowel were identified, along with sigmoid colon diverticulosis. Purulent and enteric material was found in the pericecal region. A perforated ileal diverticulum measuring approximately 5 mm was identified as the source of peritoneal contamination (Figure 3). The affected ileal segment showed inflammatory changes related to diverticulitis and perforation; however, no gross ischemia or necrosis was observed. The affected bowel segment was isolated with atraumatic non-crushing intestinal clamps, and segmental resection of approximately 50 cm of the ileum was performed. The mesentery was divided and ligated with absorbable sutures. An end ileostomy was created and matured to the skin using interrupted absorbable sutures. The affected segment involved the distal ileum, approximately 40 cm proximal to the ileocecal valve. The remaining small bowel length was approximately 230 cm and appeared viable, with adequate perfusion and no evidence of additional perforation, ischemia, or necrosis. Apart from sigmoid colon diverticulosis, no additional acute intra-abdominal abnormalities were observed.

**Figure 4:**
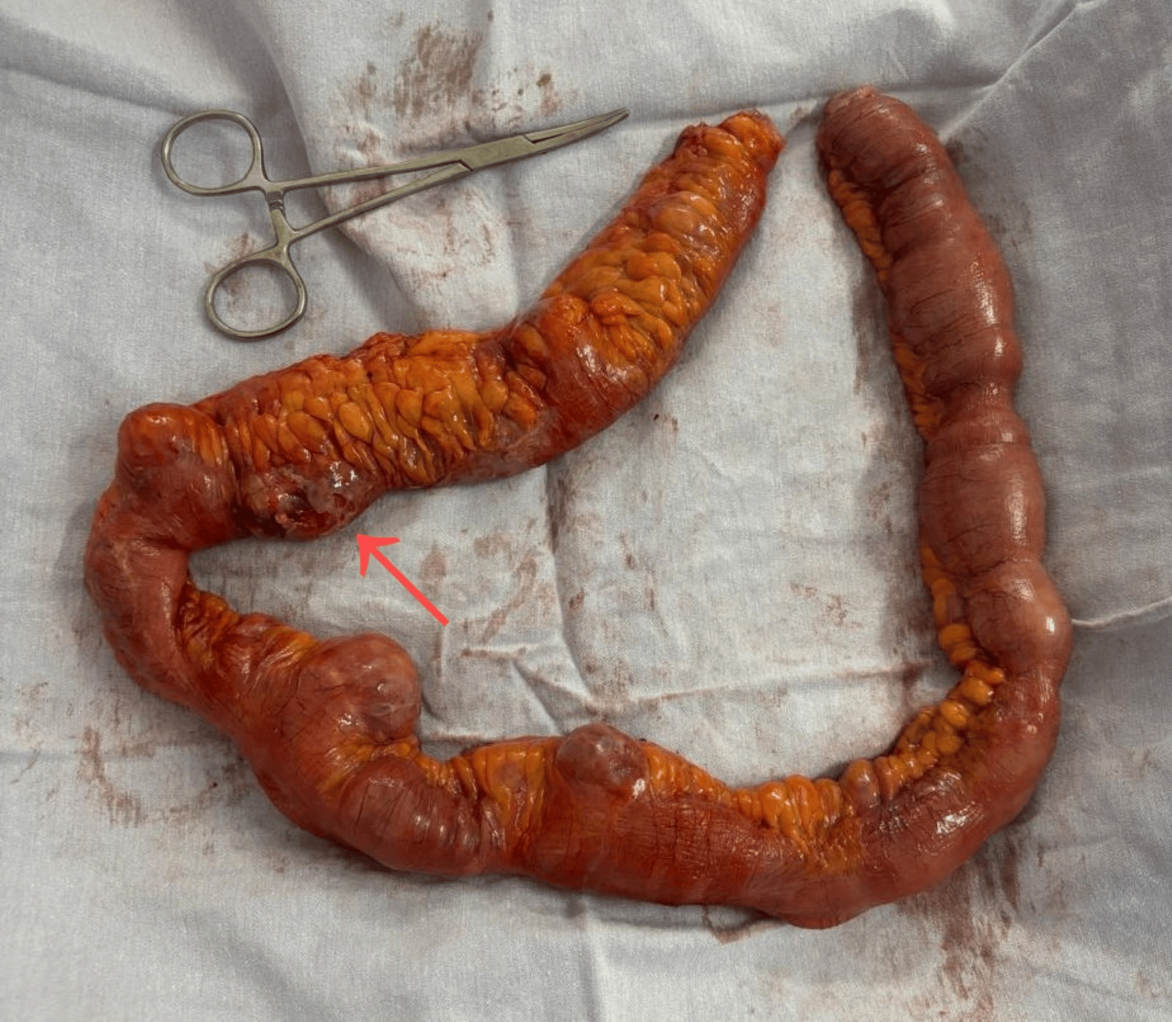
Resected ileal segment with diverticular perforation. Gross specimen of the resected ileal segment demonstrating multiple diverticula and a focal perforation site, indicated by the arrow. Approximately 50 cm of the ileum was resected because of extensive diverticular disease complicated by perforation and peritoneal contamination.

Gross examination of the resected specimen showed multiple ileal diverticula and a focal perforation site corresponding to the intraoperative findings (Figure 4). Histopathological examination revealed diverticular disease with acute diverticulitis and a perforated diverticulum measuring 5 × 5 mm. Microscopic findings included crypt abscess formation, muscular layer hypertrophy, and transmural inflammatory infiltrate. No evidence of malignancy was identified.

Postoperatively, the patient received intravenous ceftriaxone and metronidazole, proton pump inhibitor therapy, and thromboprophylaxis with enoxaparin. His clinical course was favorable; he remained hemodynamically stable, with adequate drain output and progressive improvement of inflammatory markers, including a decrease in C-reactive protein from 417 mg/L on admission to 45 mg/L on postoperative day 4. The drain was removed on postoperative day 5. Postoperative wound management consisted of routine wound care and daily wound surveillance during hospitalization. At the time of discharge on postoperative day 7, the patient was clinically stable, tolerating oral intake, with adequate ileostomy output, and the surgical wound was clean, dry, and intact, without erythema, discharge, dehiscence, or signs of infection.

At the two-week follow-up visit, the patient reported good tolerance to oral intake and adequate ileostomy output. Physical examination revealed a soft, non-tender abdomen without signs of peritoneal irritation. The ileostomy was viable and functioning properly, with no evidence of local complications. Skin sutures were removed without incident.

## Discussion

Ileal diverticulitis is an uncommon cause of acute abdomen, and perforation represents one of its most severe complications. Although diverticular disease is most frequently encountered in the colon, non-Meckel small-bowel diverticula may become clinically significant when complicated by inflammation, abscess formation, obstruction, perforation, or peritonitis. Terminal ileal diverticular perforation has been described as a rare surgical condition that may be difficult to diagnose before exploration because its clinical presentation often overlaps with more common intra-abdominal emergencies [6]. Terminal ileal diverticulosis may also remain clinically silent until an inflammatory or perforating complication occurs, which further contributes to delayed recognition [7]. In the present case, the patient presented with diffuse abdominal pain, intolerance to oral intake, absence of bowel movements and passage of flatus, markedly elevated inflammatory markers, and signs of peritoneal irritation, supporting complicated intra-abdominal infection rather than uncomplicated ileal diverticulitis.

CT was useful for narrowing the preoperative differential diagnosis in this case. Ileal diverticular disease may present as an acute abdomen and can mimic more common conditions, including acute appendicitis, terminal ileitis, Crohn's disease, infectious enteritis, colonic diverticulitis, bowel obstruction, or perforated viscus from another source [8]. Uncomplicated ileal diverticulitis may present with localized abdominal pain and inflammatory changes, whereas complicated disease may be associated with pneumoperitoneum, fluid collection, peritonitis, or the need for operative management [9]. In our patient, CT demonstrated multiple ileal diverticula, segmental bowel wall thickening, surrounding inflammatory changes, pneumoperitoneum, and an adjacent fluid collection, supporting the diagnosis of perforated ileal diverticulitis with secondary peritonitis. Reports of perforated or complicated jejunal diverticulitis further illustrate the broader spectrum of non-Meckel small-bowel diverticular disease and the diagnostic difficulty associated with these uncommon entities [10-13].

Management should be individualized according to clinical stability, imaging findings, disease extent, and the presence or absence of peritonitis. Selected patients with localized inflammation, contained perforation, or absence of diffuse peritoneal contamination may be candidates for conservative treatment with antibiotics, bowel rest, and close monitoring [14]. However, surgery is indicated when pneumoperitoneum, diffuse peritoneal signs, enteric contamination, or clinical deterioration is present. In this patient, urgent exploratory laparotomy was justified by CT evidence of perforation, clinical peritonitis, and intraoperative purulent and enteric contamination. Segmental resection was chosen because the perforation occurred within an ileal segment affected by multiple diverticula, making local repair less suitable due to the risk of leaving diseased bowel in place. In complicated non-Meckel small-bowel diverticular perforation, resection of the affected segment is commonly performed to achieve source control and remove the diseased bowel [15,16]. Creation of an end ileostomy was also appropriate given the emergency setting, advanced age, enteropurulent contamination, and need to reduce the risk of anastomotic complications.

Histopathological examination confirmed diverticular disease with acute diverticulitis, crypt abscess formation, muscular layer hypertrophy, transmural inflammation, and a perforated diverticulum, without evidence of malignancy. This confirmation was clinically relevant because perforated terminal ileal diverticular disease may be difficult to distinguish from other inflammatory, ischemic, infectious, or neoplastic processes before definitive evaluation [6]. Therefore, histopathological assessment remains important not only to confirm the diagnosis of diverticulitis but also to exclude alternative conditions that may influence postoperative follow-up and long-term management [16].

This case highlights the importance of considering ileal diverticulitis in elderly patients with acute abdomen and signs of peritonitis, particularly when CT demonstrates ileal diverticula with adjacent inflammatory changes, pneumoperitoneum, or localized fluid collection. Although the absence of bowel movements and passage of flatus may suggest obstruction, CT did not show mechanical obstruction or ischemia in this patient, and the main pathological event was perforation with secondary peritonitis. This distinction is clinically relevant because ileal diverticulitis can occasionally present with obstructive complications, as described in previous reports [17]. Early recognition, appropriate imaging interpretation, and timely operative management are essential to achieve source control in patients with complicated ileal diverticulitis.

## Conclusions

Perforated ileal diverticulitis is a rare but important cause of acute abdomen, particularly in elderly patients with nonspecific symptoms and signs of peritonitis. Contrast-enhanced CT is valuable for identifying ileal diverticula, associated inflammatory changes, pneumoperitoneum, and localized fluid collections, which may support early diagnosis before surgery. In patients with perforation, enteric contamination, or secondary peritonitis, timely operative management is essential for source control. This case highlights the importance of considering ileal diverticulitis in the differential diagnosis of acute peritonitis and supports the segmental resection of the affected bowel as an effective treatment when complicated disease is present.
